# Summary of the proceedings of the International Forum 2018: “Value-based radiology”

**DOI:** 10.1186/s13244-019-0717-7

**Published:** 2019-03-15

**Authors:** European Society of Radiology (ESR), Michael Fuchsjäger, E. Lee, V. M. Rao, J. A. Brink, B. Gonzalez Ulloa, J. M. Lozano Barriga, C. Homsi, M. Á. Pinochet Tejos, T. El-Diasty, K. Mohanan, W. Lee, H. Honda, D. Varma, L. Lawler, L. E. Derchi, R. García-Mónaco

**Affiliations:** European Society of Radiology (ESR), Am Gestade 1, 1010 Vienna, Austria

**Keywords:** Value-based radiology, Healthcare system, Transition, Cost, patient, Outcomes

## Abstract

The International Forum, established by the European Society of Radiology (ESR), aims to discuss important topics in the field of radiology with radiological partner societies from outside Europe. Value-based healthcare is currently a hot topic around the world and has been addressed in many papers. The ESR chose the topic “value-based radiology” for the ESR International Forum at ECR 2018 to discuss the trend within radiology to move from volume-based to value-based practice. The value-based healthcare (VBH) concept defined “value” as health outcomes achieved for patients relative to the costs of achieving them (European Society of Radiology (ESR) 2017, Insights Imaging 8: 566). Value should increase the health outcome and decrease the cost of delivering the outcomes. Radiology is simply counted as a cost and therefore, it is important that the radiologists themselves have an active role in the transition to be recognised as clinicians taking care of the patients.

## Main messages

- Value-based healthcare defines value as patient outcomes over costs.
- The value provided by radiology is going beyond the volume of services delivered.
- Radiologists should have an active role in the transition.
- It is important to show that radiologists play an active role in patient care.

## Introduction

The ESR established the ESR International Forum, formerly known as the International Summit, in order to discuss important radiological topics with international societies from outside Europe. The ESR International Forum is held yearly at the European Congress of Radiology (ECR) and participation is by invitation only. Previous topics included the relation between radiology and nuclear medicine, the position of ultrasound in radiology, the relation of general radiology and subspecialty radiology, the implementation of clinical decision support and imaging referral guidelines in the clinical routine, and the position of interventional radiology within radiology [1].

The following societies were invited to deliver a presentation and to present the point of view of their respective country or region: The American College of Radiology (ACR), the Asian Oceanian Society of Radiology (AOSR), the Canadian Association of Radiologists (CAR), the Chinese Society of Radiology (CSR), the Colombian Association of Radiology (ACR), the Egyptian Society of Radiology and Nuclear Medicine (ESRNM), the Indian Radiological and Imaging Association (IRIA), the Inter-American College of Radiology (CIR), the International Society of Radiology (ISR), the Japan Radiological Society (JRS), the Korean Society of Radiology (KSR), the Mexican Federation of Radiology and Imaging (FMRI), the Mexican Society of Radiology and Imaging (SMRI), the Radiological Society of North America (RSNA), the Radiology Society of the Emirates (RSE), the Royal Australian and New Zealand College of Radiologists (RANZCR), and the Radiological and Diagnostic Imaging Society of Sao Paulo (SPR). The host presented the situation in Europe. In addition, representatives of non-European past “ESR meets” countries of the last five years were invited to the meeting.

### The situation in North America

*E. Lee*, representing the **Canadian Association of Radiologists (CAR)**, presented the baseline/primer document “The value of radiology in Canada” written by the Conference Board of Canada. The Conference Board of Canada is the largest non-partisan, not-for-profit, evidence-based research organisation in Canada dedicated to building a better future for Canadians by making their economy and society more dynamic and competitive [2]. As it is important to demonstrate to lawmakers and policymakers that radiology adds value to the health system, the primer document serves the purpose of informing the CAR and provincial work on the subject. The CAR on the other hand has the ability to reach audiences such as policymakers, lawmakers, patient groups and the public, in addition to radiologists and allied health professionals. The primer document may be used by provincial bodies for their own cumulative work on the subject. One of the main goals of the report is to demonstrate how radiology adds value to a health system that is under pressure. E. Lee presented figures on the international comparison of health spending and the total health expenditures in Canada (1975-2014) from the 2015 Naylor Report on Innovation in Health Care. The Naylor report provides a lot of the background about the pressures and challenges facing the healthcare system, and shows the areas that should be targeted for research in order to determine where and how radiology adds value. Pressures are related to expenditure, human resource strain, wait times or lack of efficiency gain, for example. The Conference Board Report, as mentioned, is intended as a primer and uses three examples: breast cancer screening, teleradiology, and interventional neuroradiology. These were the case studies that helped to flesh out the meso-level narrative between the macroeconomic pressures and microeconomic experience of the average Canadian patient or community. The report also provides a framework for additional research projects to examine the value of radiology from different perspectives. Future projects should use this primer as a baseline for further research. The framework takes into account economic and societal benefits. Finally, E. Lee presented infographics that have been widely circulated across Canada and shall enable radiologists to be seen more, heard more, and understood better.

*V.M. Rao* presented for the **Radiological Society of North America (RSNA)**, describing the aims and ideas of value-based radiology as follows: Inappropriate studies and variation in the performance of studies should be reduced. There are many unnecessary interventions; MRI utilisation almost doubled in the period from 2000 to 2008, but after the rapid growth it has stabilised and is trending down. Radiologists should take full responsibility for managing the imaging of patients in order to add value for the patients. After patients schedule their exams, they should be encouraged to visit RSNA’s website radiologyinfo.org to educate themselves about the procedures. Radiologists should contact patients and talk to them at the follow-up or when requested, make the whole procedure patient friendly, offer lower cost programmes and navigate through the complexity of payment as the payment system is very complicated. Furthermore, radiologists can add value for referring physicians by having daily consultants in every subspecialty of the department, embed reading rooms in the specialty clinics of referring physicians, and improve the reports themselves. Radiologists can improve their reports by, for instance, adding their contact information at the bottom of their reports, and encouraging referring doctors and patients to contact them with any questions. All reports should be made available to patients via a portal after a short embargo period and structured using standardised terminology following RSNA’s Radlex and Structured Reporting Initiative. Patients need to realise that radiologists are taking care of them. Finally, it is very important to show the linkage between diagnosis and treatment also in non-radiology literature which receives the attention of policymakers.

*J.A. Brink*, on behalf of the **American College of Radiology (ACR),** talked about the ACR’s Imaging 3.0 initiative that has been popularised by B. Allen, Chair of the ACR Board of Chancellors 2014-2016. The initiative is a roadmap to transition the practice of radiology from volume- to value-based, from transactional to consultative, from radiologist centred to patient centred, from interpretation focused to outcomes focused, from commoditised to integral, and from invisible to accountable imaging care. The initiative addresses issues such as payment systems, patients, and radiology relevance, and shall leverage information technology (IT) such as data mining, structured reporting, clinical decision support (CDS) and communication tools, to achieve these changes. Radiologists need to enhance and own the entire patient experience in radiological care to ensure their relevance to the clinical team. Radiologists should be responsible for all aspects of imaging care, i.e. order, report and communicating wisely. Regarding ordering, the appropriate use of imaging is crucial in terms of over-diagnosis and under-diagnosis, disease detection, and population health. In this regard, clinical decision support (CDS) systems, patient education, and appropriate screening and biomarker research are relevant. Concerning reporting wisely, J.A. Brink mentioned ACRassist, a programme that shall ease actionable reporting. Structured reporting and evidence-based follow-up recommendations are pivotal in terms of communicating wisely.

### The situation in Latin America

*B. Gonzales Ulloa* presented on behalf of the **Mexican Federation of Radiology and Imaging (FMRI)**, an apolitical association that integrates the different radiological colleges and radiological societies at national level. The FMRI is working on the integral development of the associated radiologists, promoting scientific meetings, congresses, continuing education and training, covering all specialty areas, as well as promoting and unifying Mexican radiology. B. Gonzales presented the structure of the Mexican healthcare sector. Value-based imaging has been implemented in Joint Commission certified hospitals. Some of the private imaging centres have also implemented certain metrics to evaluate the performance of radiologists. Public hospitals, on the other hand, continue measuring the performance of radiology units based on the number of exams carried out in a given period. The quality of images, their diagnostic value, and the associated costs vs benefits are poorly evaluated. In the private sector, insurance companies only reimburse procedures and radiological exams if the reports are carried out by certified radiologists. Radiology service providers, paid by insurance companies, are implementing the image-based value criterion as they must comply with the policies of insurance companies. In Mexico, there are differences between private medicine and public healthcare providers, which makes the implementation of metrics to assess the value-based image difficult, as the key performance evaluation in the public sector is based only on the number of image studies per period. In summary, value-based imaging can be found only in private medicine in Joint Commission certified hospitals and some radiological centres. The metrics to assess value-based imaging are not yet standardised. The insurance companies are key players in this regard as they optimise the costs, improve cost-benefit analysis, and require compulsory participation of certified radiologists.

*J.M. Lozano Barriga*, representing the **Colombian Association of Radiology (ACR)**, presented the situation in Colombia. He started his presentation by providing some general information about the ACR and the health system in Colombia. J.M. Lozano Barriga pointed out that the radiologists are frequently considered as machines that produce imaging examinations; radiology is simply considered and measured as a cost whereas the diagnosis has not been regarded as the first important result. The volume-based system is important in Colombia as there are few radiologists, insurers and hospitals; therefore, radiologists need to attend many patients. The volume-based system produces low visibility of the radiologist to the patient though, as physicians request many inappropriate or unnecessary studies, as the government and insurance companies are interested in a greater number of patients for a greater economic income without interest in quality. Value-based radiology will enhance the radiologists’ importance in the delivery of health care services focusing on quality, safety, appropriateness, and efficiency. Although it seems very difficult to switch from a volume- to a value-based system in Colombia at the moment, it is more than necessary to begin to work on changing the paradigms and start the process of changing the system. J.M. Lozano Barriga presented some activities and programmes that the ACR is developing and working on with regard to value-based radiology.

*C. Homsi*, on behalf of the **Radiological and Diagnostic Imaging Society of Sao Paulo (SPR),** presented background information on the society, which is an affiliate of the national society Brazilian College of Radiology and Diagnostic Imaging (CBR). The SPR organises the biggest congress in Latin America. There are initial governmental and private actions directed to value-based imaging in Brazil. The CBR works through its Radiation Protection Committee and its accreditation programme. The SPR strongly supported the creation of LatinSafe which is an initiative that was launched in 2015 and advocates for radiation protection for patients. It is directed to radiologists, physicians in general as well as the lay public. The private system services have approximately 15 million people supported by the payment model known as fee-for-service which rewards the volume and complexity of exams and procedures. There are occasional experiences of pay for performance with a tendency to follow this path. The public healthcare system is well designed but marked by low financing and ineffective administration.

*M.Á. Pinochet Tejos* presented on behalf of the **Interamerican College of Radiology (CIR)**. In Latin America, the value equation has been discussed by health managers and administrators in public and private sectors during the last decade. However, the priority of the region continues to be access and coverage, while equality is still a pending task in most of the countries. Latin America is geographically very large with big cities, small towns and rural areas combined with different socioeconomic realities and thus, a difference in acquisition and access to the newest technology. The major investment and most important technological developments are in the private sector. Diagnostic imaging follows two paths, i.e. a public sector trying to give more coverage and a private sector focusing on better quality of service. Results of a multi-country study on value-based healthcare which included Brazil, Chile, Colombia and Mexico were presented. The four countries showed different levels of progress for value-based healthcare with three of them having low cohesion to the principles of value-based healthcare and only Colombia obtained a moderate score. Nevertheless, the study shows important examples of efforts made by the four countries as a clear indication that they are implementing critical elements of value-based healthcare. Although evidence shows value-based care and value-based imaging are the best methods for lowering healthcare costs while increasing quality care, moving from a fee-for-service to a fee-for-value system will take more time in Latin America than in other geographical areas, due to the region still being in a transitional stage of striving to reach access and coverage. Considering the countries’ investment in health, the transition has proved to be more difficult than expected. There is no formal evidence of the incorporation and progress of value-based imaging in Latin America, therefore, it is necessary for CIR to promote continuous innovation to improve results and reduce costs and promote personalised and patient-centred care that builds a good experience for the patient in imaging, among other areas.

### The situation in Egypt

*T. El-Diasty*, on behalf of the **Egyptian Society of Radiology and Nuclear Medicine (ESRNM)**, spoke about the situation in Egypt. The ESRNM aimed at establishing a value-based radiology (VBR) programme in response to developments in Egyptian healthcare systems in general, and the trend within radiology to move from volume- to value-based practice in particular. The ESRNM strongly believes VBR is a necessary complement to existing VBH concepts. The society is determined to establish a VBR programme to help Egyptian radiologists deal with changes in the evolution from volume- to value-based evaluation of radiological activities. The whole organisation has to change in a value-based healthcare environment [3]. A key enabler of value-based imaging is also to embrace a culture of continuous quality improvement (CQI) that ensures both qualitative and quantitative methods to assess quality. Value-based imaging needs a core culture of CQI in safety, performance, appropriateness, and outcomes [4]. Barriers to VBR in Egypt are the questions of need, lack of training, and time. In regions that do not have enough residents, it is difficult to use VBR methods in isolation. Other specialties have addressed this by working in small groups and making the problem-solving process part of continuing medical education or continuous quality improvement. T. El-Diasty presented the achievements so far, namely integration of training on management, leadership, and health economics in the training agenda, and assigning an ESRNM director of radiation protection, Dr. Dina Husseiny, for international and regional cooperation and effective national implementation to ensure patient's safety and use of imaging guidelines. Further, the ESRNM adopted the ESR European imaging referral guidelines. He also pointed out the challenges such as limited funding and financial constraints of ESRNM, the large number of radiologists and huge number of medical facilities and the fact that ESRNM so far cannot support research among radiologists to investigate value due to limited funding.

### The situation in India

*M. Kunnumal*, on behalf of the **Indian Radiological and Imaging Association (IRIA)**, presented some background information on the association. IRIA aims to educate practicing radiologists about the latest developments in the field of radiology and imaging. IRIA through its academic wing “Indian College of Radiology & Imaging” organises continuing medical education (CME) programmes to educate residents and young radiologists. In order to promote radiological study and practice and imaging, the Indian Radiological and Imaging Association organises annual conferences, which provide an unparalleled platform for educational and academic exchange. Each year thousands of medical and science professionals from across the country attend the annual conference of IRIA. India is a large country and there are not enough radiologists. The situation is highly inhomogeneous as there are hospitals with the best facilities on the one hand and on the other hand villages with no access to imaging. M. Kunnumal stressed that the radiologist should be seen as a clinician who interacts with the patient, which already happens with regard to ultrasound. Interaction with other clinical colleagues is crucial, as is striving for minimal imaging and maximum benefit. The communication of imaging results is pivotal and should be in a clinically useful format. Relevant differentials are essential as well and the correlation with clinical and other investigations should be included in the work of radiologists.

### The situation in Korea and Japan

*W. Lee* presented on behalf of the **Korean Society of Radiology (KSR).** W. Lee provided background information on the society and the Korean Congress of Radiology (KCR). Value should increase the health outcome and decrease the cost of delivering the outcomes. Value goes beyond imaging interpretation and focuses on the outcome and safety of the patient. A more efficient medical system is needed in an ageing society which results in more chronic diseases. There is new technology available which in turn results in higher medical costs. The healthcare system in Korea is government operated. The health care satisfaction is very high in Korea, even known as the highest in the world. The reimbursement for medical imaging has been better than for other medical practices. W. Lee presented data from the OECD (Organisation for Economic Co-operation and Development) Indicators from 2015 on healthcare resources in Korea as well as numbers of CT scans; the number of CT scans is increasing very steeply. In addition, the new Korean government wants to expand the health insurance benefit coverage. The volume of medical practice will thus be further increased, and the quality can thus not be guaranteed. The Korean government therefore increased the budget for assessing and increasing medical quality from 100 million USD in 2015 to 700 million USD in 2018. Further, the reimbursement model shall be different, changing from cost-based reimbursement to performance-based. The Korean Society of Radiology is preparing for the new healthcare system that should be value-based and where patients’ safety matters. The cost-effectiveness in medical practice will increase, which will result in quality and value.

*H. Honda* presented on behalf of the **Japan Radiological Society (JRS)**. In the past 6 years, the national healthcare expenditure (NHE) rapidly increased in Japan. The number of examinations in various MRI hospitals is very high. Therefore, a better distribution of resources is required. H. Honda presented the results of a survey on appropriate imaging utilisation in Japan that was conducted among accredited radiology training hospitals. The aim was to survey whether imaging is being performed appropriately and if radiologists intervene if a request is deemed inappropriate or ambiguous. The results show that legal risk plays a major role in this regard; even if a request is not recommended, physicians order it and radiologists perform it to avoid the risk of missing a disease. The JRS launched Japan Safe Radiology, a nationwide and government supported project to improve medical techniques in terms of safety, standardisation and optimisation of images and apply it to medical policy. The project targets six topics related to safety and efficiency of medical imaging: appropriate distribution of scanners and radiologists, appropriate maintenance of scanners, radiation dose management, standardisation as well as optimisation of image quality and radiology report, and appropriate utilisation of imaging [5]. In terms of appropriate utilisation, a clinical decision support system will probably be the key point for value-based radiology. The JRS is planning on adding a seventh topic to the project, namely value-based radiology. Value-based imaging must be considered in relation with the diagnosis and treatment of the patient. In addition, insurance and legal support should be required.

### The situation in the Asia-Oceania region

*D. Varma* presented for the **Asian Oceanian Society of Radiology (AOSR).** The practice of value-based radiology is very diverse in the Asian Oceanian region. In most countries radiology continues to be a service provider and the clinical role of the radiologist has not evolved. The key indicator is still turnaround times (TAT) of reports. At the Malaysian College of Radiologists, the term value-based imaging is not used, nor is the term volume metrics in the evaluation of radiology services. Measurements are taken from customer satisfaction surveys and doctor’s feedback/surveys in private and government hospitals. These surveys address issues to ensure radiology continues to provide value. D. Varma further presented Choosing Wisely Australia, an initiative to reduce unnecessary tests, treatments, and procedures for patients in hospitals [6]. The Choosing Wisely in Australia 2017 Report shows that only one-quarter (28%) of 2,500 consumers surveyed agreed that having a medical test when it was not needed could be harmful to their health. In a separate survey, around half of general practitioners (47%) and specialists (55%) strongly agreed they had a responsibility to help reduce the inappropriate use of tests, treatments and procedures. Choosing Wisely Australia is clinician-led, aims to improve safety and quality in healthcare by eliminating unnecessary tests, treatments and procedures, and it is part of a global initiative running in almost 20 countries. The initiative is aimed at significantly changing the mindsets and behaviours of health professionals and patients and to successfully challenge the notion that “more is better” when it comes to managing a person’s health [7].

### The situation in Australia and New Zealand

*L. Lawler*, presented on behalf of the **Royal Australian and New Zealand College of Radiologists (RANZCR)**. He presented some background information on RANZCR. It is important to be aware of the meaning of value to the referrers, who want accurate confident advice when they need it, to the patients who want convenient service and to feel cared for, and to the funders who want less wastage and better outcomes. In this regard, RANZCR is building closer working relationships with sister colleges and organisations, and is providing world class training, education, and CME. In addition, RANZCR is including lay people on the Board and committees. L. Lawler presented InsideRadiology, which is a free, advertising-free resource that provides information in plain English for patients and their families, as well as clinical information for referrers and health professionals. It relies on members to not only write content, but also assist in the multidisciplinary editorial process and counts over 140,000 visits per month taking into account local and international use. RANZCR developed a suite of educational modules to improve the appropriateness of referrals for medical imaging. The modules aim to influence how health professionals think about the place of imaging in patient assessment and care and to provide evidence-based clinical decision rules (CDRs). These include a set of cases for several clinical scenarios and incorporate the use of evidence-based decision support tools for appropriate referrals for imaging, including topics such as clinical decision rules, adult head trauma, and paediatric ankle trauma, among others [7]. These topics formed the basis of RANZCR’s contribution to the Choosing Wisely initiative in both Australia and New Zealand.

### The situation in Europe

*L.E. Derchi* presented on behalf of the **European Society of Radiology (ESR)**. In many European countries, the organisation of healthcare relies on a national health system that acts as facilitator, organiser and payer, and provides care to citizens. National systems are inhomogeneous and differ in terms of organisation, governance and means of funding and payment. The concept of value-based healthcare, however, is being discussed also in Europe, and a few experimental implementations of the system have already been initiated [3]. L.E. Derchi mentioned the ValueBased HealthCare Center Europe that manages several private hospitals throughout Europe, and there has been an attempt to manage the Karolinska Institute using this method. The ESR published a concept paper on value-based radiology in *Insights into Imaging*^1^ in 2017 in response to trends in healthcare that increasingly emphasise value-based aspects in relation to quality of care, patient safety, and reimbursement systems. Value-based healthcare concepts focus on “outcomes” as the cornerstone for evaluating healthcare processes, cost effectiveness, and healthcare professionals’ performance. What has been lacking so far is the inclusion of the diagnostic process, which constitutes the integral first part of every care cycle. The ESR argues that the diagnosis should be recognised as the first ‘outcome’ for patients [8]. Metrics are proposed for measuring the quality of radiologists’ diagnoses and the various ways in which radiologists provide value to patients, other medical specialists, and healthcare systems at large [3]. L.E. Derchi concluded by stating that it is crucial to work both within the radiology community to ensure a high quality of the diagnoses (the first outcome in each patient’s episode of care) and with all other clinicians to be able to demonstrate and measure the real impact of radiology on the final patient outcome.

### Position of the International Society of Radiology (ISR)

*R. García-Mónaco*, on behalf of the **International Society of Radiology (ISR),** presented the ISR’s mission to facilitate the global endeavours of the ISR’s member organisations to improve patient care through medical imaging and acts as a consultant for the World Health Organization (WHO). He emphasised that value depends on the results of care obtained rather than the volume of services delivered. In the *New England Journal of Medicine* article ’What is Value in Health Care’ by M.E. Porter, medical imaging is not even considered in the value-based healthcare concept. The ISR has not addressed specifically the subject of VBR, however, improving medical education is among the ISR’s main goals. The ISR is mainly devoted to help or act in emerging areas of the world in order not to overlap with national or continental societies’ endeavours. As a global facilitator the ISR could assist if required by members or by the WHO [1]. R. García-Mónaco stressed that value in any profession is a precious asset. It provides an opportunity for radiologists to get patient recognition of their work as a key integrating player in modern health care.

## Conclusion

Value-based healthcare aims to reduce costs whilst improving quality of the healthcare system in general. In terms of value-based radiology, one of the main aims is to show how radiology adds value to healthcare.

The Conference Board of Canada produced a primer document ‘The value of radiology in Canada’ to inform the CAR on this subject that in turn can reach out to policymakers, lawmakers as well as patients. The ACR’s Imaging 3.0 initiative in the United States, a roadmap to transition from volume- to value-based practice, addresses payment systems and patients, for example.

In Latin America, value-based imaging can mainly be found in private sectors. In Colombia, for example, there is a lack of radiologists, therefore, the volume-based system continues to be important. Latin America, being a geographically large region with big cities on the one hand and rural areas on the other hand, has no formal evidence of incorporating value-based radiology. Nevertheless, radiological societies themselves are developing and working on activities and programmes in this regard. LatinSafe, for instance, is dedicated to education in radiation protection and Japan Safe Radiology targets a diverse range of topics related to safety and efficiency of medical imaging.

In Egypt as well, there is determination to work on a programme which helps the radiologists in the country to move from volume-based to value-based practice. In Korea, the government increased the budget for assessing and increasing medical quality and the society embraces the value-based healthcare system. Regarding the Asian Oceanian region, in most countries radiology is seen primarily as a service provider whereas the role of the radiologist has not evolved. Choosing Wisely Australia is aiming to improve the quality of healthcare and is implemented in Australia’s leading health services to reduce unnecessary tests. RANZCR created InsideRadiology where patients as well as referring physicians can inform themselves on clinical radiology tests, treatments and procedures. According to IRIA (India), radiologists should be seen as clinicians, who interact with patients.

In Europe, some experimental implementations have already been initiated. ValueBased HealthCare Center Europe, an association promoting the concept of value-based healthcare, manages several private hospitals throughout Europe. Furthermore, the ESR has established a Working Group on Value-Based Imaging (as of July 2018: Subcommittee on Value-Based Radiology) that produced a concept paper on value-based radiology elaborating on the concept, metrics, and perspective.

The ISR considers value as a precious asset that helps radiologists to receive the patients’ recognition and to be seen as doctors who play a key role in patient care and contribute to the treatment plan.

To sum up, actions have already been taken in some parts of the globe to move from volume-based to value-based radiology, whereas in others volume-based radiology continues to be the main practice. Nonetheless, there was consensus that value-based radiology is the path to be followed in the near future.
